# Prevalence of Self Induced Abortion by Self-Administration of Abortive Pills among Abortion-related Admissions in a Tertiary Care Centre

**DOI:** 10.31729/jnma.5287

**Published:** 2020-12-31

**Authors:** Bibechan Thapa, Nisha Sharma, Yam Prasad Dwa

**Affiliations:** 1 Department of Gynaecology and Obstetrics, KIST Medical College and Teaching Hospital, Mahalaxmi-1, Lalitpur, Nepal

**Keywords:** *abortion (induced)*, *complications*, *self-administration*

## Abstract

**Introduction:**

Each year, unsafe medical abortion costs the lives of thousands worldwide. Despite the legalization of abortion in Nepal in 2002, many still seek services from unauthorized sources. This has led to grave consequences including death. Our objective is to find out the prevalence of self-induced abortion by self-administration of abortive pills and related complications.

**Methods:**

It is a descriptive cross-sectional study carried out among abortion-related admissions in a tertiary care centre from June 15, 2018 to March 15, 2020. Ethical approval was taken from the institutional review committee (076/077/51). Data was collected using pre-designed profoma and analysed in Statistical Package for the Social Sciences version 26. Point estimate at 95% Confidence Interval (CI) was calculated along with frequency and proportion for binary data.

**Results:**

Out of 223 cases enrolled, 37 (16.6%) (9.6-23.6 at 95% Confidence Interval) were self-induced abortion cases by self-administration of abortion pills. The mean gestational age at time of intake of pills was 7+^6^±3+^1^ week of gestation. The majority were diagnosed with incomplete abortion 14 (37.8%) followed by septic abortion 8 (21.6%). A surgical evacuation was performed in 25 (67.6%). Anaemia was observed in 19 (51.3%) with severe anaemia in 4 (10.8%). Blood transfusion was carried out in 14 (37.8%). Post abortive contraception was accepted by only 16 (42.3%).

**Conclusions:**

Medical abortion is safe if done under supervision but self-induced abortion by self-administration of abortion pills has high complications rate. Therefore, further studies exploring different dimension of the serious issue is need of time.

## INTRODUCTION

As of 2010-2014, an estimated 55.9 million abortions occur each year globally.^1^ At least 8% of maternal deaths occur worldwide from unsafe abortion with around 22,800 women dying yearly from its complications. Almost all abortion-related deaths occur in developing countries, with the highest being in Africa.^2^ In Nepal abortion was legalized in 2002, and comprehensive services were made available in 2004.^3^ Still many women today are receiving unsafe services from unauthorized providers.

Medical abortion with mifepristone and misoprostol is safe for termination of pregnancy up to 63 days if practiced under medical supervision.^4^ Despite clear guidelines, due to easy and illegal accessibility, many women self-administer these drugs. Some consider it as a method of birth spacing and depend on it without knowing its complications ranging from severe haemorrhage to death.^5^

Therefore, this study aims to find the prevalence of self-induced abortion following self-administration of abortive pills in a tertiary care center.

## METHODS

This study is a descriptive cross-sectional study was done in patients diagnosed with abortion from June 15 2018 to March 15 2020 at KIST Medical College & Teaching Hospital. Ethical approval was taken from the institutional review committee (076/077/51). All case records of in-patients mentioning women admitted with complications following self-administration of abortion pills were included while cases with complications following medical abortion done in government-accredited authorized center and service provider were excluded from the study. Data was retrieved by reviewing patients' records from the medical records department and was entered into a self-designed proforma.

Convenience sampling was done and the sample size (n) was calculated as,

$$\begin{array}{ll} \text{n} & \text{= }{\text{Z}}^{\text{2}}\times \text{p}\times \text{(1}-\text{p)}/{\text{e}}^{\text{2}}\\ & \text{= }{\left(1.96\right)}^{\text{2}}\times 0.5\times 0.5/{(\text{0.07)}}^{\text{2}}\\ & \text{= }196 \end{array}$$

Where,

n = required sample size
Z = 1.96 at 95% Confidence Interval (CI)
p = prevalence, 50%
e = margin of error, 7%

The data was entered and analysis was carried out through Statistical Package for the Social Sciences (SPSS) version 26. The data was collected throughout the study period to meet the sample size for the study.

## RESULTS

There were a total of 223 abortion cases admitted for abortion-related complications in the study period of 21 months. Out of them, 37 (16.6%) were self-induced abortion cases done by self-administration of abortion pills. Most of them were married; 33 (89.2%) and in the 20-25 years age group; 18 (48.7%) (Table 1).

Out of 37 patients, 25 (67.6%) presented to the emergency room while 12 (32.4%) presented to out patient department. Per vaginal bleeding 30 (81%) was the most common presenting symptom followed by pain abdomen 29 (78.3%). There was a history of the passage of fleshy mass in 17 (45.9%) patients and per vaginal discharge in 5 (13.5%). Seven (18.9%) complained of dizziness, 5 (13.5%) had a fever, 3 (8.1%) presented with loss of consciousness while 1 (2.7%) patient also had a history of abnormal body movement. The duration of symptoms ranged from 1 day to 30 days with a mean of 8.4±8.2 days.

**Table 1:** Demographic profile of patients.

Variables	Frequency n (%)
Age group	
<20 yrs	3 (8.1)
20-25 yrs	18 (48.7)
26-30 yrs	13 (35.1)
31-35 yrs	1 (2.7)
36-40 yrs	1 (2.7)
>40 yrs	1 (2.7)
^*^Ethnicity	
Disadvantaged Janajatis (Magar, Tamang, Rai, Lama, Tharu)	18 (48.6)
Disadvantaged non-Dalit Terai caste group (Yadav, Sohar, Takur)	4 (10.8)
Religious Minorities (Muslim)	1 (2.7)
Relatively advantaged Janajatis (Newar, Gurung)	3 (8.1)
Upper caste group (Brahmin, Chhetri)	11 (29.7)
Marital Status	
Married	33 (89.2)
Unmarried	4 (10.8)

* Ethnics codes as defined by Health Management Information system 2013

Gestational age (GA) has been calculated from last menstrual period using Naegele's formula. It is found that abortive medication was used as early as 30 days (4 +2 weeks of Gestation (WOG)) and as late as 114 days (16+2 WOG). Mean GA at the time of self-administration of abortive medication is 48.3±22 days (7+6±3+1 WOG). Mean GA at the presentation after an abortive attempt to our facility was 64.5±20.99 days (9+2±3 WOG) with a patient presenting as early as at 37 days (5+2 WOG) GA and as late as at (17+4 WOG) GA. The total duration from abortion attempts to presentation was a minimum of 1 day and a maximum of 52 days with a mean of 12.83±13.2 days.

Seven (18.9%) cases have a previous history of induced abortion. Out of them, five (13.5%) had a history of one previous induced abortion while two (5.4%) had a history of two induced abortions. Most abortions were attempted before 9 weeks of gestational age in 27 (79.97%) cases (Table 2).

**Table 2:** Reproductive health information of the participants.

Gestational age at the time of abortion attempt	Frequency n (%)
<9 weeks	27 (72.97)
9-12 weeks	8(21.62)
>12 weeks	2 (5.41)
Gravidity	
Primigravida	14 (37.8)
Multigravida	23 (62.2)
Gravida 2	6 (26)
Gravida 3	11 (47.8)
Gravida 4	4 (17.4)
Gravida 5	2 (8.6)

On examination, minimum systolic blood pressure was 80 mmHg and the maximum was 130 mmHg with a mean of 102±13.3 mmHg. Likewise, the minimum mean arterial pressure was 60 mmHg, the maximum was 96.7 mmHg with a mean of 77.8.±10.7 mmHg. Pallor was present in 15 (40.5%) patients. Hemoglobin level measured showed a minimum of 5.3 gm/dl and a maximum of 13.5 gm/dl with a mean value of 10.45±2.36 mg/dl. Fourteen (37.8%) received blood transfusion (Table 3).

**Table 3:** Anemia and blood transfusion profile.

Anemia	Frequency n (%)
Mild (9-10.9)	9 (24.3)
Moderate (7-8.9)	6 (16.2)
Severe <7	4 (10.8)
No anemia >10.9	18 (48.6)
**Blood Transfusion**	
1 pint	3(21.4)
2 pints	6 (42.9)
3 pints	3(21.4)
4 pints	2 (14.3)

Majority of women 37.8% (14) were diagnosed with incomplete abortion followed by septic abortion which was present in 21.6% (8). Five (13.5%) patients were diagnosed with ectopic pregnancy of which one was very rare case of ovarian molar ectopic pregnancy (Table 4).

**Table 4:** Diagnosis and complications.

Diagnosis	Frequency n (%)
Incomplete Abortion (1 incomplete abortion with UTI)	14 (37.8)
Ectopic pregnancy (2-Ruptured Ectopic, 1 Tubal abortion with a ruptured ovarian cyst, 1 Ovarian Molar Pregnancy, 1 unruptured tubal ectopic pregnancy)	5 (13.5)
Septic Abortion	8(21.6)
Complete Abortion (1 Complete abortion with UTI)	3 (8.1)
Missed abortion	3 (8.1)
Failed MA (live pregnancy)	2 (5.4)
Inevitable abortion	1 (2.7)
Incomplete Abortion with H. Mole	1 (2.7)

The total duration of hospital stay was a minimum of 1 day and a maximum of 7 days with a mean of 2.97±1.44 days. The minimum duration from last pregnancy was 5 months and the maximum duration was 132 months with mean 38.4±34.1 months.

All the patients received antibiotic treatment. None of the patients needed critical care admission or inotropic support. Post abortive contraception counselling was provided to everyone but only 16 (43.2%) accepted it (Table 5).

**Table 5:** Management modality.

Management	Frequency n (%)
MVA (Manual Vacuum Aspiration)	24 (64.9)
Conservative	6 (16.2)
Laparotomy	4 (10.8)
MI (Medical Induction)	2 (5.3)
Suction & Evacuation	1 (2.7)
**Acceptance of Post-abortive Contraception rate: 43.2%**	
**Type of Contraception Method**	**Frequency n (%)**
Depo Provera	8 (50%)
Implants (Jadellae)	7 (43.8%)
IUCD	1 (6.2%)

## DISCUSSION

In 21 months, 37 patients presented with various complications following self-administration of abortive pills. The minimum age of the women was 17 years and the maximum was 42 years with a mean age of 25.3±5.36 years. The majority (83.8%) were from 20-30 year of age and those greater than 30 years of age were approx 8%. However, in a study conducted by Giri et al.^6^ 52% of women were of 20-29-year age group and 44% were from the 30-39 year age group.

In our study 37.8% were primigravida and 62.2% were multigravida. Out of the total, 16.2% were Gravida 2, and 45.9% were Gravida 3 or more including Gravida 5 (2). The percentage of Primigravida in our study was higher in comparison to other studies. In the study done by Giri et al.^6^ 79% of women were multigravida and 21% were primigravida, a similar finding was seen in K. Nivedita et al.^7^ In the study conducted by Singh M et al.^8^ 10% were primigravida, 15% were gravida 2, 75% were gravida 3 or more including 17% of the total who were more than gravida 5.

Gestational age (GA) was calculated from the last menstrual period (LMP) using Naegele's formula. It was found that abortive medication was used as early as 30 days and as late as 114 days. Mean GA at the time of self-administration of abortive medication is 48.3±22 days. The majority (70.27%) of the women had self-administered abortive pills within approved GA of fewer than 9 weeks It was similar to other studies by Giri et al.^6^ and Jethani M et al.^9^ which was 60%. At 9 to 12 WOG, 18.9% of women had taken abortive medication while 5.4% had taken medication beyond 12 WOG. In the study conducted by Giri et al.^6^ 60% of women had consumed abortion pills within approved nine weeks gestation while 19% had consumed after nine weeks and 21% after twelve weeks.

The unmarried women belonged to the age group 20-24 years and constituted 10.8% in our study. It was close to the study done by K. Nivedita et al.^7^ where 12.5% were unmarried but contrary to our study unmarried age group was 15-19 years.

In our study, 94.6% of patients were admitted for 1-5 days. The maximum duration of hospital stay in our study was of 7 days. In a study conducted by K. Nivedita et al,^7^ duration of hospital stay was 1-5 days which was 75% and 1 patient had a hospital stay of more than 10 days.

In our study, 37.8% of women were admitted with a diagnosis of incomplete abortion which was less in comparison to a study conducted by Giri et al.^6^ where 60% of cases were of incomplete abortion. Jethani M et al.^9^ reported 57.45%, Goyal N et al.^10^ reported 75%, and K. Nivedita et al.^7^ reported 70% of cases with an incomplete abortion. Septic abortion comprised of 21.6% in our study. On the contrary, a study done by K. Nivedita et al.^7^ reported 7.5% cases with septic abortion, Giri et al.^6^ showed 6.5%, Jethani M et al.^9^ reported 4.3% and Goyal N et al.^10^ showed 5% patients with sepsis.

In our study, ectopic pregnancy was diagnosed in 13.5% of pregnancy which is higher when compared to other studies, which shows 6.5% in Giri et al.^6^ 1.06% in Jethani M et al.^9^ and 2% in Goyal N et al.^10^

In our study failed medical abortion was observed in 5.4%. Near similar finding was observed in a study conducted by Jethani M et al.^9^ where 6.3% of women had live continued pregnancy. Other studies were done by Goyal N et al.^10^ had 3%, Giri et al.^6^ reported 13% continued pregnancy and K Nivedita et al.^7^ reported a 22.5% failed abortion with continued live pregnancy. In our study two pregnancies, 5.4% had molar pregnancy which had been rarely reported in self-induced abortion.

Surgical evacuation (MVA and Suction & evacuation) was performed in 67.8% of patients. Finding was nearly similar to Giri et al.^6^ which showed 71% and Singh M et al.^8^ which showed 73%. Other management modalities included conservative management which was done in 16.2% (tab misoprostol in incomplete abortion, antibiotics in septic abortion, general measure incomplete abortion), laparotomy was performed in all patients with ectopic pregnancy i.e. 10.8%.

In our study anemia was present in 51.4% of which severe anemia was present in 10.8 %. Near about findings were reported by Jethani M et al.^9^ with anemia in 57.45% of which 11.7 % was severe anemia and K. Nivedita et al.^7^ with anemia in 50% with severe anemia in 12.5%. However, in the study conducted by Giri et al.^6^ anemia was present in 79% of patients and 37.5% of the patient had severe anemia.

Blood transfusion was received by 37.8% in our study. Giri et al.^6^ reported blood transfusion needed in 52%, Jethani M et al.^9^ reported 20.21% patients, and K Nivedita et al.^7^ blood transfusion was needed in 15% of the patients. Most of the patients i.e 42.9% required a two-pint blood transfusion in our study while Giri et al.^6^ showed 29%. In our study, 13.5% of patients required three pints or more of blood transfusion. In a study conducted by Giri et al.^6^ 22.9% required three or more pints of blood transfusion.

Post abortive contraception was accepted by 43.2% of whom 50% received Depo Provera, 43.8% Jadellae, and 6.2% received IUCD. In the study conducted by Singh M et al.^8^ contraceptive counseling was done in all patients during their hospital stay, and patients accepted contraceptives in the hospital itself in the form of OC pill, IUCD, DMPA injection, CHHAYA, condoms, and tubal ligation was accepted by 16% cases.

## CONCLUSIONS

Safe abortion services are every women's reproductive rights. Medical Abortion is safe if done under supervision by authorized personal in authorized institution following authorized standard protocols. But self-induced abortion done by self-administration of abortion pills has high complications rate. Severity of complications is too high to underestimate yet less has been explored and known about this issue. Developing countries like Nepal has high prevalence rate of Self-induced abortion done by self-administration of abortion pills, this implies to multifactorial causation like lack of awareness of abortion services and abortion laws, lack of awareness on contraception and family planning, associated social stigma, easy availability of abortion pills over the counter and many more.

Therefore, studies exploring different dimension of the serious issue is need of time.
